# Effect of Harvest Time on Non-Volatile Metabolites in Japonica Rice

**DOI:** 10.3390/foods14071224

**Published:** 2025-03-31

**Authors:** Mengnan Teng, Xiaoting Xing, Pengli Jiang, Xiaoliang Duan, Dong Zhang, Hui Sun, Chunfang Zhao, Xingquan Liu, Zhigang Yao, Motonobu Kawano

**Affiliations:** 1 Academy of National Food and Strategic Reserves Administration, Beijing 100037, China; tmn15968339133@163.com (M.T.); xxt@ags.ac.cn (X.X.); dxl@ags.ac.cn (X.D.); sh@ags.ac.cn (H.S.); 2 College of Food and Health, Zhejiang A&F University, Hangzhou 311302, China; liuxq@zafu.edu.cn; 3 Tibet Autonomous Region Food and Strategic Reserves Administration, Grain & Oil Ctr Lab, Lhasa 850032, China; 18108909761@163.com; 4 Institute of Food Crops, Jiangsu Academy of Agricultural Science, Nanjing 210014, China; 5 Satake Manufacturing (Suzhou) Co., Ltd., Suzhou 215129, China; zg-yao@satake.cn; 6 Satake Corporation, Hiroshima 739-8602, Japan; m-kawano@satake-japan.co.jp

**Keywords:** japonica rice (*Oryza Sativa* L.), harvest time, non-volatile metabolites, liquid chromatography-mass spectrometry (LC-MS)

## Abstract

A large number of non-volatile metabolites are produced during the growth of rice; however, few studies have focused on the changes in these metabolites at different harvest times. In this study, Nangeng 5718 (a rice variety) was taken as the research object to study the changes in rice metabolites at different harvest times. Liquid chromatography mass spectrometry (LC-MS) was used to analyze the non-targeted metabolomics of rice at different harvest times in Nanjing, Huai’an, and Lianyungang in the Jiangsu Province of China. The results showed that 2111 metabolites were annotated by the human metabolome database (HMDB), accounting for 94.96% of the total number of metabolites. Rice metabolites included one categories, including 312 fatty acyls, 275 organooxygen compounds, 261 carboxylic acids and derivatives, etc. The results of the Kyoto encyclopedia of genes and genomes (KEGG) pathway showed that autophagy–other, ABC transporters, and glycerophospholipid metabolism had a great effect on rice heading to harvest. The experiments showed that L-histidine in Nangeng 5718 was upregulated. These results provide comprehensive insights into the relationship between rice harvest time and changes in metabolites.

## 1. Introduction

Japonica rice is one of the most important grain crops globally, serving as a major source of carbohydrates and essential nutrients [1]. Over 90% of rice consumption and production occurs in Asia [2]. Rice plants are usually harvested by farmers at the end of wax maturity to the beginning of maturity [3]. At present, research on the impact of harvest time on rice has focused on rice yield and specific quality indicators. Wang et al. [4] found that the harvest period had a significant effect on the dry matter accumulation of rice. With the extension of the harvest time, the dry matter loss of the two cultivars increased first and then decreased. Xangsayasane et al. [5] found that broken rice increased greatly, while head rice decreased sharply when the harvest was delayed to 35 and 45 days after 75% flowering.

Metabolomics is an emerging tool used to study the final products of gene expression with a low molecular weight (<1000 Da) in living organisms and investigate changes in metabolites [6]. It usually studies the changes in the metabolic response of all small molecule metabolites (amino acids, sugar alcohols, lipids, etc.) in biological systems under different growth environments, periods, and external stimuli [7]. In order to adapt to the changing environment, rice produces a large number of metabolites to meet its own needs during the growth process. The chemical composition of rice determines its taste and nutritional value to a large extent, and is an important basis for evaluating its quality. Huang et al. [8] proved that the degradation and oxidation of phospholipids during the storage process of brown rice accelerated the deterioration of its quality. Long et al. [9] found the protein content was negatively correlated with taste and cooking quality, and a higher protein content usually led to poor rice quality. Jin et al. [10] stated rice varieties with a higher amylose content had a harder texture and lower eating and cooking qualities. Additionally, rice quality traits are also affected by changes in starch, proteins, lipids, and interactions among them under environmental stresses [11,12].

At present, there are many studies on metabolites in the growth process of rice, mainly focusing on seedlings, booting, and grain filling. However, there are relatively few studies on the changes in metabolites during the rice harvest stage. Wang et al. [13] found rice was most sensitive to low-temperature stress at the booting stage (LTB); LTB significantly increased the activity of ornithine-δ-aminotransferase and proline content in the leaves, leading to increased albumin and protein contents in the grains. Qu et al. [14] showed the metabolism of lipids, sugars, and phenolics was essential for the development of the pollen wall. Liang et al. [15] stated high temperature during grain filling enhanced aspartic acid metabolism in grains and led to the accumulation of amino acids and protein components of the aspartate family, and HT may improve the nutritional quality of rice during grain filling. Non-volatile substances including carbohydrates (starch), proteins, and lipids are closely related to the quality of rice.

The harvest time is a key factor affecting the content of non-volatile metabolites in rice. Harvesting too early leads to a not fully matured grain and the insufficient accumulation of substances such as sugars, starch, and amino acids, which caused the insufficient fullness of the seeds, poor taste, and low nutritional value. Harvesting too late leads to a decrease in taste quality and nutritional value [16,17]. The study of rice metabolites can intuitively analyze the differences of rice at different harvest times from a new perspective, providing a new method for determining its harvest time.

Nangeng 5718 is one of the most popular rice varieties in China and has been widely cultivated in Jiangsu Province. Nangeng 5718 has the characteristics of high yield and excellent taste quality [18,19]. Nanjing, Huai’an, and Lianyungang are all located in Jiangsu Province of China. The climatic conditions in the three regions are suitable for the growth of Nangeng 5718. In this study, UHPLC-Q Exactive HF-X MS was used to determine the non-volatile metabolites of Nangeng 5718 at different harvest times. Multivariate statistical analysis methods, such as principal component analysis (PCA) and partial least squares analysis (OPLS-DA), were used to explain the changes in the types and quantities of metabolites in rice at different harvest times. Furthermore, the key metabolic pathways involved in metabolite changes were clarified. This study provides basic insights into the changes in non-volatile metabolites during rice growth.

## 2. Materials and Methods

### 2.1. Chemical and Reagents

Rice samples (cv. Nangeng 5718) were selected from Nanjing, Huai’an, and Lianyungang of Jiangsu Province in China [20]. Paddy rice was harvested at 50, 55, and 60 days after full heading. Each harvest time consists of 3 parallels. Ten rice plants were mixed together as one sample. Methanol, acetonitrile, and water were purchased from Fisher Chemical (Shanghai, China). Formic acid was purchased from CNW (Shanghai, China). 2-Propanol was purchased from Merck (Shanghai, China). 2-Chloro-L-phenylalanine was purchased from Adamas-beta (Shanghai, China).

### 2.2. Rice Sample Preparation

The milling of brown rice was based on our previously published research [21]. A total of 1 kg of paddy rice was weighed and hulled by a JDMZ100 hulling machine (Beijing Dongfu Jiuheng Instrument Technology Co., Ltd., Beijing, China) to obtain brown rice. The brown rice was ground into brown rice flour by a KN195 kniftec knife type sample mill (Foss Analytical Instrument Co., Ltd, Beijing, China). All untreated and treated samples were stored at 4 °C for non-volatile metabolite analyses.

### 2.3. Non-Volatile Metabolite Analysis

Non-volatile metabolites were analyzed following the procedure reported by Hang et al. [22], with slight modifications. A total of 50 ± 5 mg brown rice flour was transferred into centrifuge tubes and a 6 mm diameter grinding bead was placed inside. Then, a 400 µL of methanol: water = 4:1 (v:v) extraction solution, containing four internal standards (L-2-chloroalanine (0.02 mg/mL), etc.), was added to the centrifuge tubes. Frozen tissue was ground by a grinder for 6 min (−10 °C, 50 Hz). After standing at −20 °C for 30 min, the samples were centrifuged for 15 min (13,000× *g*, 4 °C), and the supernatant was pipetted into an injection vial with an inner tube for analysis. In addition, 20 μL of supernatant was pipetted separately from each sample and mixed into a single sample for quality control (QC).

The qualitative analysis of non-volatile metabolites was performed using ultra-performance liquid chromatography tandem Fourier transform mass spectrometry system (UHPLC-Q Exactive HF-X, Thermo Scientific, Waltham, MA, USA). ACQUITY UPLC HSS T3 (100 mm × 2.1 mm i.d., 1.8 µm; Waters, Milford, MA, USA) [18], mobile phases A (95% water + 5% acetonitrile) with 0.1% formic acid and B (47.5% acetonitrile + 47.5% isopropanol + 5% water) with 0.1% formic acid, was used to separate non-volatile metabolites in rice. The injection volume was 3 μL and the column temperature was 40 °C.

The samples were ionized by electrospray. The mass spectrometry signals were acquired in the positive and negative ion scanning modes. The scan type was 70–1050 *m*/*z*; the sheath gas flow rate was 50 arb; the aux gas flow rate was 13 arb; the heater temp was 425 °C; the capillary temp was 325 °C; and ion spray voltage was 3500 V (positive ionization mode)/−3500 V (negative ion ionization mode). The s-lens RF level was 50; the normalized collision energy levels were 20%, 40%, and 60%; and the resolutions were 60,000 full MS and 7500 MS2.

### 2.4. Statistical Analysis

Metabolomics raw data were analyzed using ProgenesisQI v3.0 (WatersCorporation, Milford, MA, USA). The MS and MS/MS mass spectrometry information was matched to metabolic databases, and the metabolites were identified. Metabolite data were processed using Excel. The Ropls (R packages) software (Version1.6.2) was used for multivariate statistical analyses, such as PCA and PLS-DA. The selection of differential metabolites (DMs) was determined based on the variable weight value (VIP) obtained by the OPLS-DA model and the *p* value of the Student’s *t* test. The screening conditions were VIP > 1 and *p* < 0.05. The metabolic pathways were annotated by aligning the DMs with the KEGG database, and the pathway enrichment analysis was performed with the Python software (Version1.0.0) package scipy.Stats (Scipy Statistics).

## 3. Results and Discussion

### 3.1. Data Quality Assessment and Statistical Analysis

**Figure 1:**
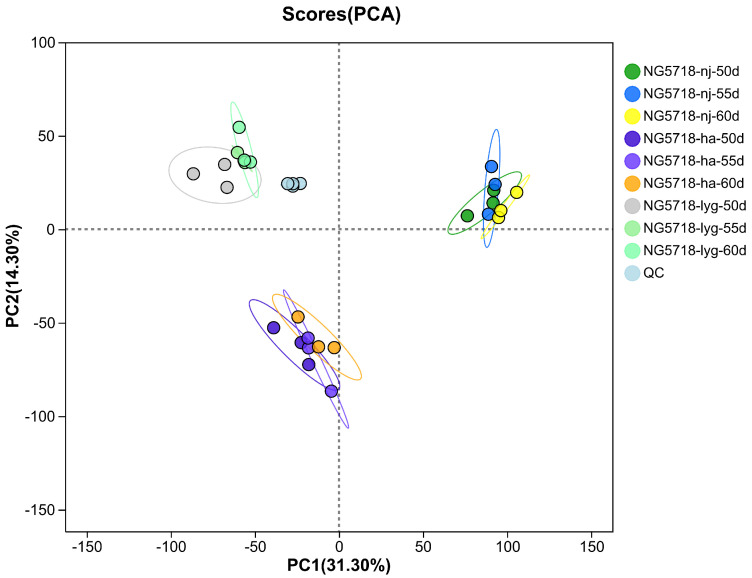
PCA score graph.

As shown in Figure 1, in both the positive and negative ion modes, the biological replicates of each group were clustered together. The results indicated that the samples in the group were highly correlated and the reproducibility of the sequencing data was reliable. The results indicated that the correlation between the samples of different treatment groups was small, while the differences in metabolites were large. In addition, the QC samples were well grouped, demonstrating a good bioanalysis and data quality.

### 3.2. Compound Classification by HMDB

**Figure 2:**
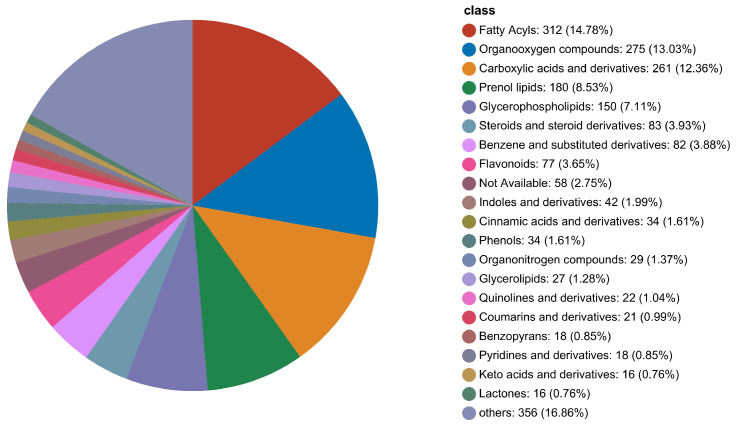
Pie chart of rice metabolite classification at different harvest times.

The metabolites obtained after data pretreatment were compared to the human metabolic database (HMDB) to obtain functional annotation information of the metabolites. In Figure 2, the number of metabolites annotated by HMDB was 2111, accounting for 94.96% of the total metabolites. Rice metabolites are divided into 21 categories, including 312 fatty acyls, 275 organooxygen compounds, 261 carboxylic acids and derivatives, 180 prenol lipids, 150 glycerophospholipids, 83 steroids and steroid derivatives, 82 benzene and substituted derivatives, 77 flavonoids, 58 not available, 42 indoles and derivatives, 34 cinnamic acids and derivatives, 34 phenols, 29 organonitrogen compounds, 27 glycerolipids, 22 quinolines and derivatives, 21 coumarins and derivatives, 18 benzopyrans, 18 pyridines and derivatives, 16 keto acids and derivatives, 16 lactones, and 356 others.

### 3.3. Screening and Analysis of Differential Metabolites

**Figure 3:**
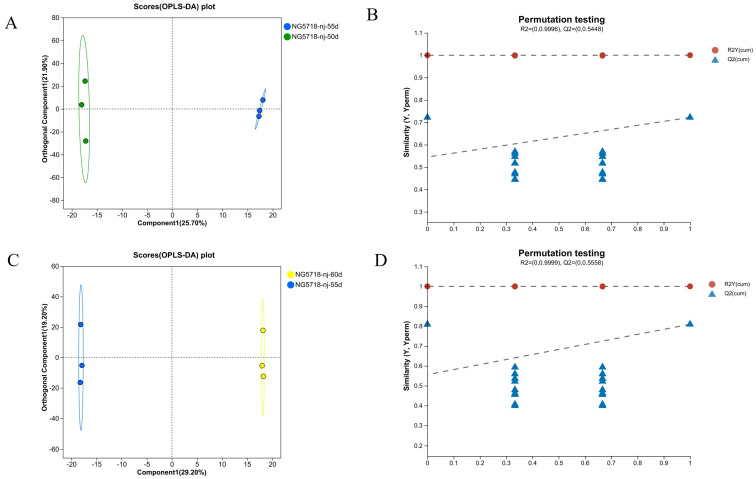
OPLS-DA score and permutation testing of Nangeng 5718 grown in Nanjing. (**A**) OPLS-DA score of 50d vs. 55d. (**B**) Permutation test of 50d vs. 55d. (**C**) OPLS-DA score of 60d vs. 55d. (**D**) Permutation test of 60d vs. 55d.

As shown in Figure 3, in the 55d vs. 50d group, the samples from the two different treatment groups were significantly separated in both the positive and negative ion modes, with obvious clustering within each group. The results showed that the metabolic profile of Nangeng 5718 was significantly different between 50 and 55 days of harvest after heading. R^2^Y and Q^2^, which represent the Y-axis interpretation rate and prediction ability of the OPLS-DA model, were both greater than 0.5. The R^2^X that represents the X-axis interpretation rate was 0.476, close to 0.5. It showed that the OPLS-DA model is stable and reliable. It has agood prediction ability. In addition, the results of the permutation testing in thepositive ion and negative ion modes showed that, with the decrease indisplacement retention, R^2^ and Q^2^ decrease. The regression line showed an upward trend. It showed that the permutation test was passed, and the model has no overfitting. The combination of the VIP value (threshold > 1) and *p* value (threshold ≤ 0.05) was used to screen the differential metabolites. Finally, 15 differential metabolites were selected for analysis. The results are shown in Table 1.

In the 60d vs. 55d group, the samples of the two different treatment groups in the positive and negative ion modes were significantly separated, and clustering within the group was obvious. The results showed that there was a significant difference in the metabolic profile of the rice harvested at 60 and 55 days after heading. R^2^Y and Q^2^ were both greater than 0.5. R^2^X was 0.485, close to 0.5. The results showed that the model is stable and reliable. It has a good prediction ability. The results of differential metabolites were shown in Table 2.

**Figure 4:**
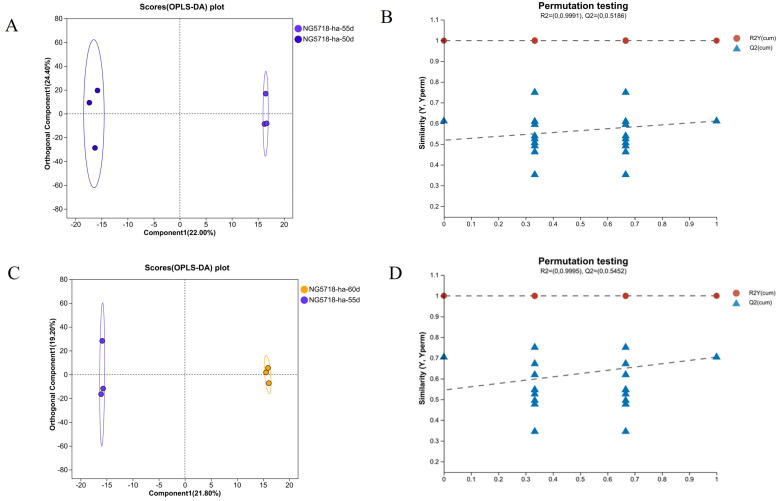
OPLS-DA score and permutation test of Nangeng 5718 grown in Huai’an. (**A**) OPLS-DA score of 50d vs. 55d. (**B**) Permutation test of 50d vs. 55d. (**C**) OPLS-DA score of 60d vs. 55d. (**D**) Permutation test of 60d vs. 55d.

**Figure 5:**
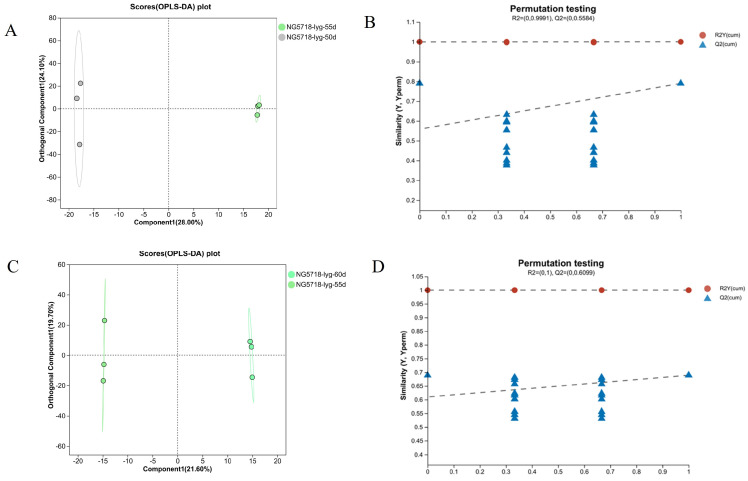
OPLS-DA score and permutation test of Nangeng 5718 grown in Lianyungang. (**A**) OPLS-DA score of 50d vs. 55d. (**B**) Permutation test of 50d vs. 55d. (**C**) OPLS-DA score of 60d vs. 55d. (**D**) Permutation test of 60d vs. 55d.

As shown in Figure 4 and Figure 5, OPLS-DA analysis and model verification displacement test were carried out for the differential grouping in Huai’an and Lianyungang areas. The results showed that there were significant differences in the metabolic levels of the different treatment groups, and the model was stable and reliable, with a good prediction ability. DMs can be screened using the VIP values obtained from the OPLS-DA analysis. The results of the differential metabolites are shown in Table 3, Table 4, Table 5 and Table 6.

**Figure 6:**
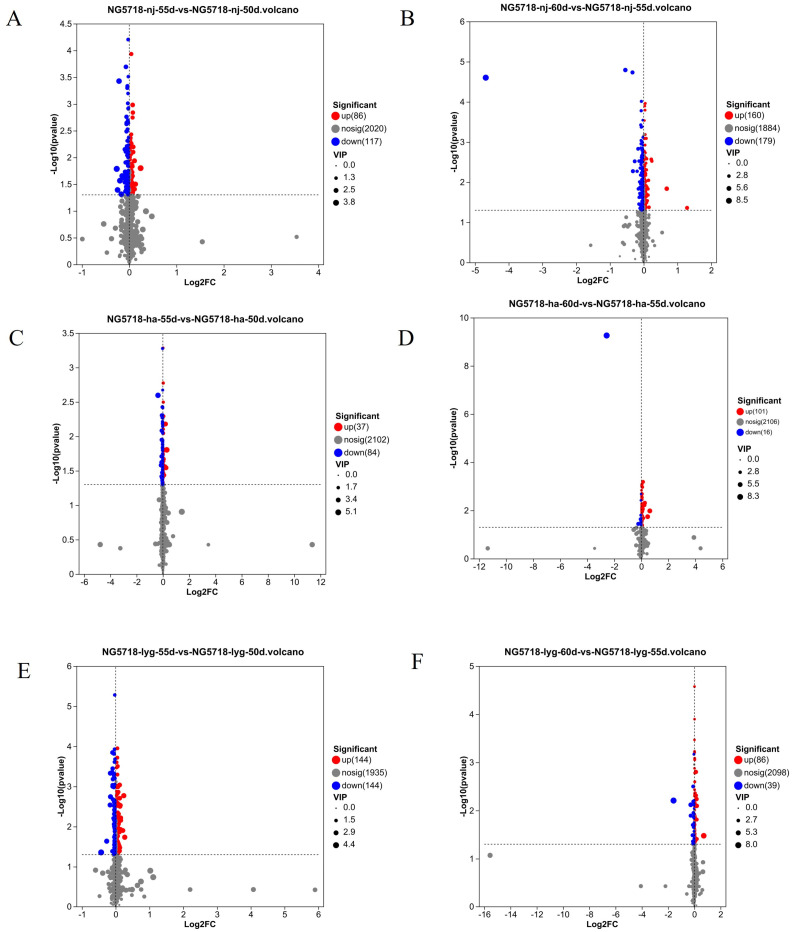
Volcanic diagram of differential metabolites. (**A**) Volcanic diagram of 50d vs. 55d in Nanjing. (**B**) Volcanic diagram of 60d vs. 55d in Nanjing. (**C**) Volcanic diagram of 50d vs. 55d in Huai’an. (**D**) Volcanic diagram of 60d vs. 55d in Huai’an. (**E**) Volcanic diagram of 50d vs. 55d in Lianyungang. (**F**) Volcanic diagram of 60d vs. 55d in Lianyungang.

VIP ≥ 1 and *p* < 0.05 were used as the criteria to screen the differential metabolites in the six differential groups, with a total of 2223 differential metabolites showing significant differences. As shown in Figure 6, there were 86 metabolites upregulated and 117 metabolites downregulated in the 55d vs. 50d group of rice grown in Nanjing. There were 160 metabolites upregulated and 179 metabolites downregulated in the 60d vs. 55d group of rice grown in Nanjing. There were 37 metabolites upregulated and 84 metabolites downregulated in the 55d vs. 50d group of rice grown in Huai’an. There were 101 metabolites upregulated and 16 metabolites downregulated in the 60d vs. 55d groups of rice grown in Huai’an. There were 144 metabolites that were upregulated and 144 metabolites that were downregulated in the 55d vs. 50d group of rice grown in Lianyungang. There were 86 metabolites upregulated and 39 metabolites downregulated in the 60d vs. 55d groups of rice grown in Lianyungang.

### 3.4. KEGG Enrichment Analysis

To clarify the functions of differential metabolites and the metabolic pathways they participate in, the differential metabolites were compared to the KEGG database for enrichment analysis. The top 20 KEGG pathways were selected for analysis.

**Figure 7:**
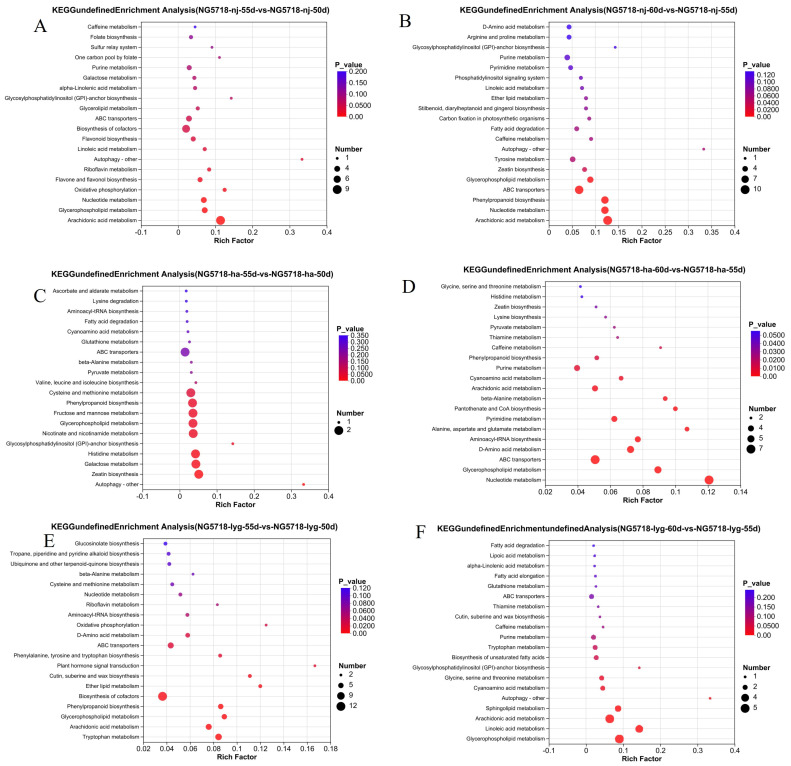
KEGG enrichment analysis. (**A**) Bubble diagram of 55d vs. 50d in Nanjing. (**B**) Bubble diagram of 60d vs. 55d in Nanjing. (**C**) Bubble diagram of 50d vs. 55d in Huai’an. (**D**) Bubble diagram of 60d vs. 55d in Huai’an. (**E**) Bubble diagram of 50d vs. 55d in Lianyungang. (**F**) Bubble diagram of 60d vs. 55d in Lianyungang.

As shown in Figure 7, in the 55d vs. 50d group of Nangeng 5718 grown in Nanjing, the autophagy–other pathway was the most enriched, with four differential metabolites identified: PE(16:1(9Z)/P-18:1(9Z)), GPEtn(16:1/20:4), PE(18:2(9Z,12Z)/18:3(9Z,12Z,15Z)), and PE(18:1(9Z)/18:3(6Z,9Z,12Z)). The number of differential metabolites enriched by the biosynthesis of cofactors was the largest. There are six main metabolites, including riboflavin, ubiquinone-2, dyspropterin, D-threo biopterin, adenosine 5′-monophosphate, and uridine-5′-monophosphate. These differential metabolites were closely related to the rice growth of Nangeng 5718 grown in Nanjing at 50 days to 55 days after heading.

In the 60d vs. 55d group of Nangeng 5718 grown in Nanjing, the autophagy–other pathway was the most enriched. This pathway enriched four differential metabolites, including PE(18:0/18:4(6Z,9Z,12Z,15Z), GPEtn(16:1/20:4), PE(18:2(9Z,12Z)/18:3(9Z,12Z,15Z)), and PE(18:3(6Z,9Z,12Z)/18:3(9Z,12Z,15Z)). The number of differential metabolites enriched by the ABC transporters was the largest. There were nine main metabolites, including adenosine, cytidine, L-alanine, L-proline, xanthosine, raffinose, inosine, cytosine deoxyribonucleoside, and myo-inositol. These differential metabolites were closely related to the rice growth of Nangeng 5718 planted in Nanjing from 55 days to 60 days after heading.

In the 55d vs. 50d group of Nangeng 5718 grown in Huai’an, the autophagy–other pathway was the most enriched. This pathway enriched three differential metabolites, including PE(16:1(9Z)/P-18:1(9Z)), PE(16:0/18:2(9Z,12Z)), and PE(18:0/20:2(11Z,14Z)). The number of differential metabolites enriched by the glycerophospholipid metabolism was the largest. There are four main metabolites, including PE(16:1(9Z)/P-18:1(9Z)), LysoPC(20:1(11Z)), PE(16:0/18:2(9Z,12Z)), and PE(18:0/20:2(11Z,14Z)). These differential metabolites were closely related to the rice growth of Nangeng 5718 planted in Huai’an from 50 days to 55 days after heading.

In the 60d vs. 55d group of Nangeng 5718 grown in Huai’an, the nucleotide metabolism pathway was the most enriched. This pathway enriched seven differential metabolites, including: uracil, guanine, uridine, cytosine, cytidine, xanthosine and adenosine 5′-monophosphate. The number of differential metabolites enriched by the ABC transporter was the largest. There were seven main metabolites, including aspartic acid, phosphate, uridine, cytidine, L-glycine, xanthosine, and L-histidine. These differential metabolites were closely related to the rice growth of Nangeng 5718 planted in Huai’an from 55 days to 60 days after heading

In the 55d vs. 50d group of Nangeng 5718 grown in Lianyungang, the plant hormone signal transduction pathway was the most enriched. This pathway enriched two differential metabolites, including indole-3-carboxaldehyde and abscisic acid. The number of differential metabolites enriched by the biosynthesis of cofactors was the largest. There were eight main metabolites, including L-methionine, nicotinic acid, riboflavin, L-tryptophan, pantothenic acid, ubiquinol (QH2), N′-formylkynurenine, porphobilinogen, S-adenosyl-l-homocysteine, adenosine, 5′-monophosphate, and D-glucuronic acid. These differential metabolites were closely related to the rice growth of Nangeng 5718 planted in Lianyungang from 50 days to 55 days after heading.

In the 60d vs. 55d group of Nangeng 5718 grown in Lianyungang, the autophagy–other pathway was the most enriched. This pathway enriched one differential metabolites, that is, PE(18:3(9Z,12Z,15Z)/20:3(5Z,8Z,11Z)). The number of differential metabolites enriched by the biosynthesis of cofactors was the largest. There were five main metabolites, including lysoPC(20:5(5Z,8Z,11Z,14Z,17Z)), PC(16:0), PC(18:2(9Z,12Z)/18:3(9Z,12Z,15Z)), PE(18:3(9Z,12Z,15Z)/20:3(5Z,8Z,11Z)), PS(15:0/22:0), and PE-NMe2(18:1(11Z)/16:0). These differential metabolites were closely related to the rice growth of Nangeng 5718 planted in Lianyungang from 55 days to 60 days after heading.

### 3.5. Metabolic Mechanism Analysis

After the KEGG enrichment analysis, we found that the differential metabolites of Nangeng 5718 were significantly aggregated in several core pathways, including autophagy–other, ABC transporters, and glycerophospholipid metabolism. Research shows that ABC transporters play a crucial role in plant growth, development, nutrient absorption, and environmental stress response [23]. ABC transporters release energy through the hydrolysis of ATP to achieve the transmembrane transport of substances, including peptides, sugars, amino acids, etc. Among them, carbohydrate and amino acid metabolism and pathways are associated with plant resistance to environmental stress [24]. Autophagy plays an important role in several stages of plant growth and development, including nutrient cycling, cellular senescence and apoptosis, and starch degradation. Huang et al. [25] showed the GW3a gene significantly affected yield-related indexes, including particle size, grain weight, and 1000-grain weight, by regulating the autophagosome pathway of starch metabolism in rice. Glycerophospholipid metabolism-related metabolites can improve the stress resistance of rice. Ma et al. [26] proved that the overexpression of TaMGD in wheat responded to high temperature stress by changing cell membrane lipid content, fatty acid unsaturation, and other factors.

Carbohydrates are an important nutrient in rice grains, which is closely related to the growth and development of rice and the taste quality of rice. With the increase in maturity, the contents of raffiseed and xylobiose showed a significant downward trend. It may be due to the decomposition of starch in rice into soluble sugars (maltotriose, xylobiose, raffinose, etc.) under environmental stress to increase the concentration of cell juice [21]. The amount of starch synthesis also directly affects the yield of rice and the degree of grain fullness. Amino acids are important flavor compounds, and changes in the type of amino acids may cause changes in the taste of rice. During the period from heading to harvest, the contents of umami and sweet amino acids, such as aspartic acid, L-histidine, and L-glutamic acid, in rice grains increased significantly, and the flavor of rice was improved [27]. The content of essential amino acids, such as L-methionine, L-threonine, and L-tryptophan, in rice grains also increased significantly, and the nutrition of rice was improved. In addition, the amino acid content in rice is closely related to its stress resistance. The metabolism of glutanine, aspartic acid, and glutamic acid is usually enhanced in rice under stress. The antioxidant capacity of rice plants is enhanced [28].

In this study, the differential metabolites enriched in each pathway in each region were analyzed. It was found that L-histidine, the key substance from rice heading to harvest, in Nangeng 5718 planted in all regions increased. When the plants were subjected to drought stress, the metabolism of sugars, proteins, and lipids accelerates. Some receptor proteins, such as histidine phosphokinase histidine kinases (HKs), sensitively captured and responded to external drought stress signals [29]. It caused changes in plant osmoregulation and antioxidant systems, and ultimately enabled the plants to adapt to drought stress or enhance their own drought resistance [30].

**Figure 8:**
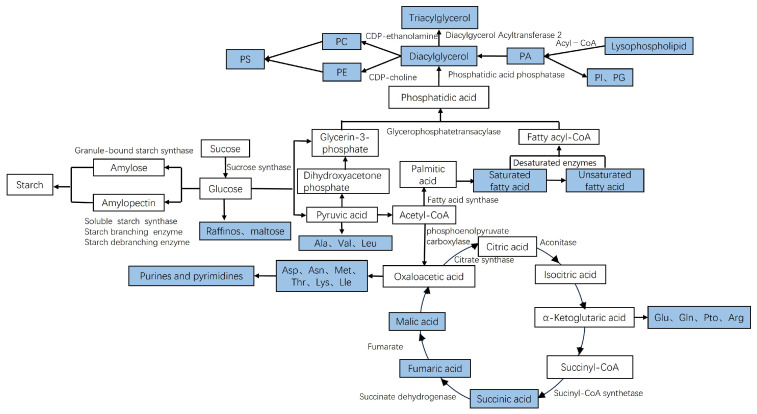
Diagram of rice growth mechanism from heading to harvest.

Grain filling is a critical process that determines the rice yield and quality, including the transportation, synthesis, and accumulation of starch, protein, and lipids [31]. As shown in Figure 8, the blue boxes indicate the detected metabolites. During the growth of rice, leaves synthesize sucrose through photosynthesis and transport it into the grains. This process provides the raw material for the synthesis of starch [32,33]. Simultaneously, carbohydrates form pyruvic acid during aerobic metabolism, which is converted into acetyl-CoA through oxidative decarboxylation, and enters the tricarboxylic acid cycle. The glucose in the grain produces a large amount of fat synthesis precursors during glycolysis, such as pyruvic acid and dihydroxyacetone phosphate. Pyruvic acid undergoes oxidative decarboxylation to form acetyl-CoA, and dihydroxyacetone phosphate produces glycerol 3-phosphate. Among them, acetyl-CoA is the raw material for the synthesis of fatty acids, and then the synthesis of various lipids [34]. At the same time, pyruvic acid, oxaloacetic acid, and α-ketoglutarate acid can also be used as a source of carbon backbone to synthesize various amino acids catalyzed by a series of enzymes, such as glutamine synthetase, glutamate synthetase, aspartate aminotransferase, etc. [35].

## 4. Conclusions

In this study, we analyzed non-volatile metabolites of Nangeng 5718 at different harvest times in Nanjing, Huai’an, and Lianyungang. A total of 2111 metabolites were annotated by HMDB, mainly including 21 categories. Through the classification and comparison of metabolites in various regions, we found that the differential metabolites between them were mainly concentrated fatty acyl molecules, organic compounds, and carboxylic acids and their derivatives. There was an increasing trend in L-histidine in Nangeng 5718 harvested in all regions. The accumulation of L-histidine in rice plays an important role in mitigating and resisting damage caused by drought stress. Additionally, with the increase in maturity, the content of oligosaccharides, such as maltotriose, xylobiose, and raffinose, in Nangeng 5718 grains decreased, while starch synthesis increased. In addition, the amino acid content increased, which improved the nutrition and flavor of rice. Therefore, 60 days after heading was determined to be the most suitable harvesting time for Nangeng 5718 based on non-volatile metabolites in rice. By determining the optimal harvest time of rice through metabolites, it is possible to avoid losses caused by harvesting too early or late, to understand the changes in the nutritional composition of rice at different harvest times and produce rice with a high nutritional value. The relationship between metabolites and yield was analyzed to improve rice yield. Our results provide a new method for evaluating the growth and development of rice and offer a new perspective for determining the optimal harvest time. Due to the fact that the harvest time determined by metabolites was 60 days after heading, it was not possible to determine the specific metabolite that was most suitable for harvesting Nangeng 5718. This study only focused on the rice variety Nangeng 5718, and future research can be extended to different varieties of rice in different environments, so as to provide a more in-depth theoretical basis for determining the harvest time of rice.

## Figures and Tables

**Table 1:** Differential metabolite screening of Nangeng 5718 grown in Nanjing from 55d vs. 50d.

Metabolite	VIP Value	*p* Value
DG(i-16:0/0:0/20:3(6,8,11)-OH(5))	3.502	0.016
PA(i-22:0/i-16:0)	3.428	0.027
Methionyl-Asparagine	3.263	0.041
PC(18:1(9Z)-O(12,13)/18:2(9Z,12Z))	3.041	0.048
PE-NMe2(18:1(11Z)/18:1(11Z))	2.715	0.032
2-Hydroxycineol	2.491	0.012
DG(16:1(9Z)/16:0)	2.271	0.039
N-(4-Amino-2,5-diethoxyphenyl)benzamide	2.226	0.019
PE(18:1(9Z)/18:3(6Z,9Z,12Z))	2.196	0.012
Enol-phenylpyruvate	2.177	0.007
13-L-Hydroperoxylinoleic acid	2.114	0.008
1-(2-Furyl)butan-3-one	2.086	0.022
5-Hexyl-2-furanoctanoic acid	2.061	0.002
1,2-Di-(9Z,12Z,15Z-octadecatrienoyl)-3-(galactosyl-alpha-1-6-galactosyl-beta-1)-glycerol	2.031	0.022
(13E)-11a-Hydroxy-9,15-dioxoprost-13-enoic acid	1.987	0.035

DG: diacylglycerol; PA: phosphatidic acid; PC: phosphatidyl choline; PE: phosphatidylerhanolamine.

**Table 2:** Differential metabolite screening of Nangeng 5718 grown in Nanjing from 60d vs. 55d.

Metabolite	VIP Value	*p* Value
5-Phenyl-1,3-oxazinane-2,4-dione	4.502	0.015
7-Amino-4-methylcoumarin	3.524	0.044
Shikimic acid	2.842	0.003
PE(18:0/18:4(6Z,9Z,12Z,15Z))	2.679	0.042
Ergocalciferol	2.563	0.035
(S)-(+)-1-(p-Hydroxy-trans-cinnamoyl)-glycerol	2.540	0.003
Valeric acid	2.369	0.024
N-[(2R)-6,7-Dihydroxy-3-oxo-1-sulfanylheptan-2-yl]acetamide	2.347	0.010
N-Palmitoyl lysine	2.323	0.021
Glutamine-glutamate	2.310	0.004
Subaphylline	2.289	0.019
Methylarbutin	2.260	0.021
5-Megastigmen-7-yne-3,9-diol 3-glucoside	2.254	0.015
1-Octen-3-yl glucoside	2.200	0.013
N-Ethyl trans-2-cis-6-nonadienamide	2.184	0.011

PE: phosphatidylerhanolamine.

**Table 3:** Differential metabolite screening of Nangeng 5718 grown in Huai’an from 55d vs. 50d.

Metabolite	VIP Value	*p* Value
3-(Octyloxy)propan-1-amine	4.278	0.016
TG(15:0/22:6(4Z,7Z,10Z,13Z,16Z,19Z)/14:1(9Z))	3.095	0.007
1-Amino-2-methylanthraquinone	2.908	0.026
15-F2t-IsoP	2.843	0.027
DG(16:0/18:3(9Z,12Z,15Z))	2.520	0.008
Cotinine glucuronide	2.498	0.023
Ne,Ne dimethyllysine	2.316	0.005
Indole-3-acetyl-myo-inositol	2.116	0.034
Fructosyl valine	2.069	0.027
Indole-3-acetyl-tyrosine	1.983	0.023
1-O-E-Cinnamoyl-(6-arabinosylglucose)	1.821	0.005
DG(18:1(9Z)/22:6(4Z,7Z,10Z,13Z,16Z,19Z))	1.759	0.012
6-Fluoro-DL-tryptophan	1.748	0.023
1-Myristoyl-sn-Glycerol 3-Phosphate	1.741	0.044
5-(6-hydroxy-6-methyloctyl)furan-2(5H)-one	1.677	0.041

TG: triglyceride; DG: diacylglycerol.

**Table 4:** Differential metabolite screening of Nangeng 5718 grown in Huai’an from 60d vs. 55d.

Metabolite	VIP Value	*p* Value
11-Hydroxy-9-tridecenoic acid	8.283	5.481
Glucomannan	4.936	0.018
DG(16:0/18:3(9Z,12Z,15Z))	3.169	0.001
5-Methyleriodictyol 7-[glucosyl-(1->4)-galactoside]	2.961	0.009
PC(18:4(6Z,9Z,12Z,15Z)/18:2(9Z,12Z))	2.498	0.011
Cytosine	2.324	0.001
Beta-D-glucosamine	2.280	0.003
Sn-glycero-3-phosphoethanolamine	2.271	0.002
Cytidine	2.226	0.017
LL-2,6-Diaminopimelic acid	2.176	0.043
N-Feruloylaspartic acid	2.098	0.010
1-Methylxanthine	2.051	0.020
N-(4-Amino-2,5-diethoxyphenyl)benzamide	2.023	0.026
L-Glycine	2.023	0.024
Vitamin A2	1.979	0.001

DG: diacylglycerol; PC: phosphatidyl choline.

**Table 5:** Differential metabolite screening of Nangeng 5718 grown in Lianyungang from 55d vs. 50d.

Metabolite	VIP Value	*p* Value
Glucomannan	4.197	0.044
1-(3-Methyl-2-butenoyl)-6-apiosylglucose	3.212	0.018
Desglymidodrine	3.075	0.023
(7′R,8′R)-4,7′-Epoxy-3′,5-dimethoxy-4′,9,9′-lignanetriol 9′-glucoside	3.042	0.002
N-Oleoyl asparagine	2.713	0.003
Indole-3-acetyl-tyrosine	2.621	0.000
Indole-3-acetamide	2.576	0.006
DG(18:4(6Z,9Z,12Z,15Z)/15:0)	2.502	0.002
PC(18:1(11Z)/18:1(11Z))	2.498	0.040
(3S,7E,9S)-9-Hydroxy-4,7-megastigmadien-3-one 9-glucoside	2.490	0.012
Indole-3-acetyl-beta-4-D-glucose	2.486	0.000
Indole-3-acetyl-myo-inositol	2.365	0.001
(3beta,6beta)-Furanoeremophilane-3,6-diol 6-acetate	2.336	0.003
L-Tryptophan	2.285	0.014
Oryzalide B	2.163	0.008

DG: diacylglycerol; PC: phosphatidyl choline.

**Table 6:** Differential metabolite screening of Nangeng 5718 grown in Lianyungang from 60d vs. 55d.

Metabolite	VIP Value	*p* Value
PE(18:3(9Z,12Z,15Z)/20:3(5Z,8Z,11Z))	6.753	0.033
MGDG(18:3/18:2)	4.673	0.008
DG(20:3(6,8,11)-OH(5)/16:0)	4.395	0.008
DG(18:2/18:1/0:0)	4.275	0.006
DG(15:0/22:6(4Z,7Z,10Z,13Z,16Z,19Z))	3.682	0.002
Tyrosyl-proline	3.356	0.013
2-Hydroxypyridine	3.056	0.015
DGDG(18:2/18:2)	2.998	0.003
13-L-Hydroperoxylinoleic acid	2.385	0.048
Indole-3-acetamide	2.307	0.008
Erucamide	2.189	0.014
Cyclohexaneundecanoic acid	2.165	0.022
LysoPE(0:0/20:1(11Z))	2.153	0.004
Glucoobtusifolin	1.945	0.002
Cis-p-Coumaric acid 4-[apiosyl-(1->2)-glucoside]	1.876	0.046

PE: phosphatidylerhanolamine; MGDG: monogalactosyldiacylglycerol; DG: diacylglycerol; LysoPE: lysophospholipyl ethanolamine.

## Data Availability

The data that support the findings of this study are available upon request from the corresponding author.
